# Association Between Dietary Habits and Depression: A Systematic Review

**DOI:** 10.7759/cureus.32359

**Published:** 2022-12-09

**Authors:** Ramaneshwar Selvaraj, Tharun Yadhav Selvamani, Anam Zahra, Jyothirmai Malla, Ravneet K Dhanoa, Sathish Venugopal, Shoukrie I Shoukrie, Ranim K Hamouda, Pousette Hamid

**Affiliations:** 1 Family Medicine/Internal Medicine, California Institute of Behavioral Neurosciences & Psychology, Fairfield, USA; 2 General Surgery, California Institute of Behavioral Neurosciences & Psychology, Fairfield, USA; 3 Surgery, California Institute of Behavioral Neurosciences & Psychology, Fairfield, USA; 4 Internal Medicine, California Institute of Behavioral Neurosciences & Psychology, Farfield, USA; 5 Internal Medicine, California Institute of Behavioral Neurosciences & Psychology, Fairfield, USA; 6 Neurology, California Institute of Behavioral Neurosciences & Psychology, Fairfield, USA; 7 Orthopaedics/Traumatology, California Institute of Behavioral Neurosciences & Psychology, Fairfield, USA

**Keywords:** diet, feeding habits, feeding behaviour, low mood, mood disorder, depression, dietary pattern, dietary habits

## Abstract

Depression being the psychiatric condition with the highest societal costs in industrialized nations, it is necessary to collect research information on the role of nutrition in depression in order to provide recommendations and guide future health treatment. Observance of healthy dietary practices has been linked to decreased depression symptoms; however, it is unknown whether this is attributable to some or all of the components.

The aim of this study was to conduct a systematic review of observational and systematic studies analyzing the association between dietary habits and depression in adolescents, adults, and older people. A variety of noncommunicable chronic illnesses and deaths have been linked to dietary and other lifestyle factors such as physical inactivity, cigarette use, and alcohol use.

Several studies have found that older people are more likely to be malnourished and depressed, which can hurt their overall health and well-being. Early in life, proper nutrition is suggested as a factor that may have a significant impact on one's mental health. It is critical to identify changes in primary care practices in order to improve the quality of life and prevent complications.

The systematic review included papers presenting human studies and published in English until January 2021, analyzing the association between dietary habits and mental health, while we studied a group of people of mixed ages. We included three systematic reviews, three cross-sectional studies, two cohorts, and one meta-analysis. According to the findings of our systematic review of observational studies, observational evidence suggests that both following a healthy diet, in particular incorporating vegetables and fruits, and avoiding a pro-inflammatory diet like junk foods, fast foods, and high meat intake may lower the risk of developing depressive symptoms or clinical depression. As a result, we recommend that the relationship between diet and depression should be investigated in prospective cohorts and randomized controlled studies that are well-designed and have more conclusive evidence regarding dietary involvement and depression.

## Introduction and background

Depression is a frequent and significant medical condition that negatively impacts your moods, thoughts, and behaviors. Fortunately, it can be treated. Depression results in emotions of sadness and/or decreased interest in formerly enjoyable activities. It can result in a number of mental and physical issues and impair your ability to perform at home and at work [1]. Depressive disorders today impose a considerable cost on the health and economy of both industrialized and developing nations. Prevalence estimates range from 3.3% to 21.4% [2].

Depression is a debilitating condition and has emerged as one of the most important issues facing global public health presently. A total of three hundred million people were predicted to be suffering from this illness in 2017, accounting for 4.4 percent of the global population at the time [3,4]. Depression and anxiety disorders cost the worldwide economy one trillion dollars a year in lost productivity, according to the World Health Organization [4].

Habitual decisions made by individuals or a group of people about what foods to consume are called dietary habits. Vitamins, minerals, carbs, proteins, and fats are all necessary components of a healthy diet. Human health is greatly influenced by a person's dietary habits and decisions. Dietary factors and other lifestyle factors such as physical inactivity, cigarette use, and alcohol use have all been linked to a variety of noncommunicable chronic illnesses (NCDs) and death [5].

Several studies have shown that elderly people are more likely to suffer from malnutrition and depression, both of which can have negative consequences on their overall health and well-being. In order to improve the quality of life and prevent complications, it is critical to identify changes in primary care practices [6]. Neurological mechanisms associated with inflammation, oxidative stress, neuroplasticity, mitochondrial function, and the gut microbiome that are altered by dietary intake may influence the likelihood of developing depression [7].

Individuals' dietary or feeding habits vary, making it difficult to determine the precise source of the diet that produces depression. Dietary habits might include the type of cuisine consumed, the time between meals, and the quality of food consumed. A range of food styles are included in the dietary habits, and it can provide an indication of whether or not a person has healthy or unhealthy food intake habits, as well as the factors that may be responsible for depressive episodes in the future. It is critical to understand the association between daily food habits and depression since it has the potential to play a significant role in the prevention of the disease in the future [8].

Although it is commonly known that depressed people tend to change their lifestyle and eating habits, there are not enough data to imply that the dietary pattern one follows in their life changes their behavior and leads to depression. However, given the numerous combinations and interactions between nutrients in a person's daily diet, investigating the relationship between specific nutrients and disease presents significant constraints. To date, it has been difficult to link differences in illness prevalence or symptoms to a specific food source because of the complexity of diet as an exposure. Dietary patterns and specific nutrient consumption may also act as confounding factors in diet-disease relationships. This has led to an increased focus on dietary habits and their role in the development of illness [9]. Hence, a broad systematic review of published articles was performed to evaluate the association between dietary habits and depression.

## Review

Method

The systemic review followed the Preferred Reporting Elements for Systematic Reviews and Meta-analysis (PRISMA) group guidelines [10].

Search Strategy

The study began on November 20, 2021, with online libraries serving as our database. For our data gathering, we used PubMed and Google Scholar.

Our search tactics for keywords and medical subject headings (MeSH) were as follows:

A systematic evaluation was conducted on peer-reviewed papers published in English from 1st January 2010 to 31st January 2021 that presented the findings of observational and systematic studies, which were included in a systematic review. They were found using the PubMed and Google Scholar databases, as well as manual searches of the reference lists from the studies that were included. Table 1 shows the search approach used to select the studies for the systematic review.

**Table 1:** Search strategy MeSH: Medical subject headings

PubMed	Dietary habits AND Feeding habits OR Feeding behaviours OR Dietary patterns OR Diet AND Depression OR Mood disorder OR Low mood OR Atypical depression OR Depletion of serotonin AND (( "Feeding Behavior/adverse effects"[Majr] OR "Feeding Behavior/analysis"[Majr] OR "Feeding Behavior/complications"[Majr] OR "Feeding Behavior/education"[Majr] OR "Feeding Behavior/prevention and control"[Majr] )) OR ( "Feeding Behavior/adverse effects"[Mesh:NoExp] OR "Feeding Behavior/analysis"[Mesh:NoExp] OR "Feeding Behavior/complications"[Mesh:NoExp] OR "Feeding Behavior/education"[Mesh:NoExp] OR "Feeding Behavior/prevention and control"[Mesh:NoExp] ) AND (( "Depression/analysis"[Majr] OR "Depression/complications"[Majr] OR "Depression/diet therapy"[Majr] OR "Depression/etiology"[Majr] OR "Depression/prevention and control"[Majr] OR "Depression/statistics and numerical data"[Majr] )) OR ( "Depression/analysis"[Mesh:NoExp] OR "Depression/complications"[Mesh:NoExp] OR "Depression/diet therapy"[Mesh:NoExp] OR "Depression/etiology"[Mesh:NoExp] OR "Depression/prevention and control"[Mesh:NoExp] OR "Depression/statistics and numerical data"[Mesh:NoExp] )
Google Scholar	Dietary habits AND Feeding habits OR Feeding behaviours OR Dietary patterns OR Diet AND Depression OR Mood disorder OR Low mood OR Atypical depression OR Depletion of serotonin

Data Extraction

Two researchers separately chose studies based on their titles, while at this stage they only removed those that may have been perceived as not meeting the inclusion criterion based on the title. Then, in the second step, these two researchers independently verified inclusion based on the abstract, excluding only those who could have been regarded as clearly not meeting the inclusion criterion without a doubt. The final assessment was conducted based on the retrieved entire text in the final stage. If there was a disagreement and the researchers could not come to a consensus after their debate, the third researcher's view was decisive.

Eligibility Criteria

There were no geographical or publication status constraints on studies that looked into the link between food habits and depression in persons. 

Inclusion Criteria

We included only full-text peer-reviewed articles which are available for free and research publications published between 2010 and 2021 were considered. We also included English language articles which included human research, groups of healthy people, and people with certain chronic or acute illnesses. We also added studies that included groups of teenagers, adults, and elderly people. We included studies that talk about regular eating patterns/intake, as well as the type and quality of food. We included articles that evaluate mental health and also articles about people who are depressed or are showing indications of depression.

Exclusion Criteria

We excluded articles that have never been published, as well as books, papers, and research articles published before 2010. We also excluded studies where the entire text is not available and studies that involve animal research, secondary research, research in the lab, duplication of databases, and a lack of detailed results. We also excluded grey literature and studies in which the evaluation of the eligibility papers is of poor quality.

After the studies were verified and included in the third stage of evaluation (based on the full text of the paper), the following information was extracted from each publication: author, country/location, study group, number of participants, gender proportions, age, dietary habits, method of outcome assessment, psychological measure, observations, and conclusions. The narrative review was written based on the provided information, and each article included in the systematic review had its data organized. Table 2 illustrates the features of the nine articles that were featured.

**Table 2:** Characteristics of the included studies MDS - The Major Depression Rating Scale

SL.NO	AUTHOR	TOPIC/STUDY	YEAR OF PUBLICATION	AGE GROUP	STUDY DESIGN	RESULTS
1	Dharmayani et al. [11]	Association between fruit and vegetable consumption and depression symptoms in young people and adults aged 15-45:	2021	15-45	Systematic Review	The effect of fruits and vegetables on decreasing the likelihood of developing depression and depressive symptoms is not conclusive.
2	Głąbska et al. [12]	Fruit and vegetables intake in adolescents and mental health	2020	Adolescents	Systematic Review	On the basis of a systematic review of observational studies, it may be concluded that consumption of fruit and vegetable products is positively associated with adolescents' mental health. Particularly beneficial for adolescents' general mental health were green vegetables, yellow vegetables, and fresh fruits.
3	Gibson-Smith et al. [13]	Association of food groups with depression and anxiety disorders.	2020	Mean Age 52 years ± 13.2	Cohort Study	Non-refined grains and, to a lesser extent, vegetable consumption and alcohol consumption appeared to be the driving variables for the association between the total MDS score and depression/anxiety. However, the effect of the entire diet on mental health remains crucial. These associations were not limited to depressive symptoms, but also included clinically diagnosed depression and anxiety disorders that had not been established previously. Those who had recovered from depression or anxiety disorders lacked the association. It should be investigated whether consuming more whole grains and vegetables can prevent or alleviate depression and anxiety.
4	Gregório et al. [14]	Dietary patterns characterized by high meat consumption are associated with other unhealthy life styles and depression symptoms	2017	>18	Cohort Study	Our findings indicate that unhealthy dietary practices (meat dietary practices) are a component of a lifestyle that includes physical inactivity, smoking, and alcohol consumption. Moreover, unhealthy dietary practices are associated with depressive symptoms.
5	Jacka et al. [15]	Association of western and traditional diets with depression and anxiety in women	2010	20 - 94	Cross Sectional Study	This is the first study to suggest that the significant effect of diet quality on common chronic noncommunicable diseases extends to mental illnesses with high prevalence. To determine the direction of the relationships and to rule out residual or unrecognized confounding, however, this relationship must be examined further in prospective studies that are well-designed.
6	Lassale et al. [16]	Healthy dietary indices and risk of depressive outcomes	2019	>15	Systematic Review and Meta-analysis	Observational evidence suggests that adhering to a healthy diet, particularly a traditional Mediterranean diet, and avoiding a pro-inflammatory diet are both associated with a decreased risk of depressive symptoms or clinical depression.
7	Quirk et al. [9]	The association between diet quality, dietary patterns and depression in adults	2013	Adults	Systematic Review	This review provides a critical summary of the current evidence concerning the relationship between diet quality and depression, a relatively new area of study. Further research is urgently needed to determine whether there are true causal relationships between diet and depression.
8	Weng et al. [17]	Is there any relationship between dietary patterns and depression and anxiety in Chinese adolescents?	2011	11 - 16	Cross Sectional Study	Adjusting for relevant confounders revealed that the snack and animal food patterns were associated with a high risk of depression and anxiety, whereas the traditional diet pattern was associated with a low risk for them. The study provided epidemiological evidence of a strong relationship between traditional, snack, and animal food diets and mental symptoms in Chinese urban adolescents.
9	Vafaei et al. [18]	Malnutrition is associated with depression in rural elderly population	2013	70.6 ± 7.3	Cross Sectional Study	According to our research, there is substantial evidence that malnutrition and depression are prevalent among the elderly and may have detrimental effects on their health and well-being. Therefore, it is essential to detect changes in primary care in order to enhance life quality and reduce complications. Nutritional interventions should only be implemented as part of a comprehensive strategy, given that malnutrition has multifactorial causes and is frequently co-occurring with multiple other problems.

Results

Literature Search

**Figure 1:**
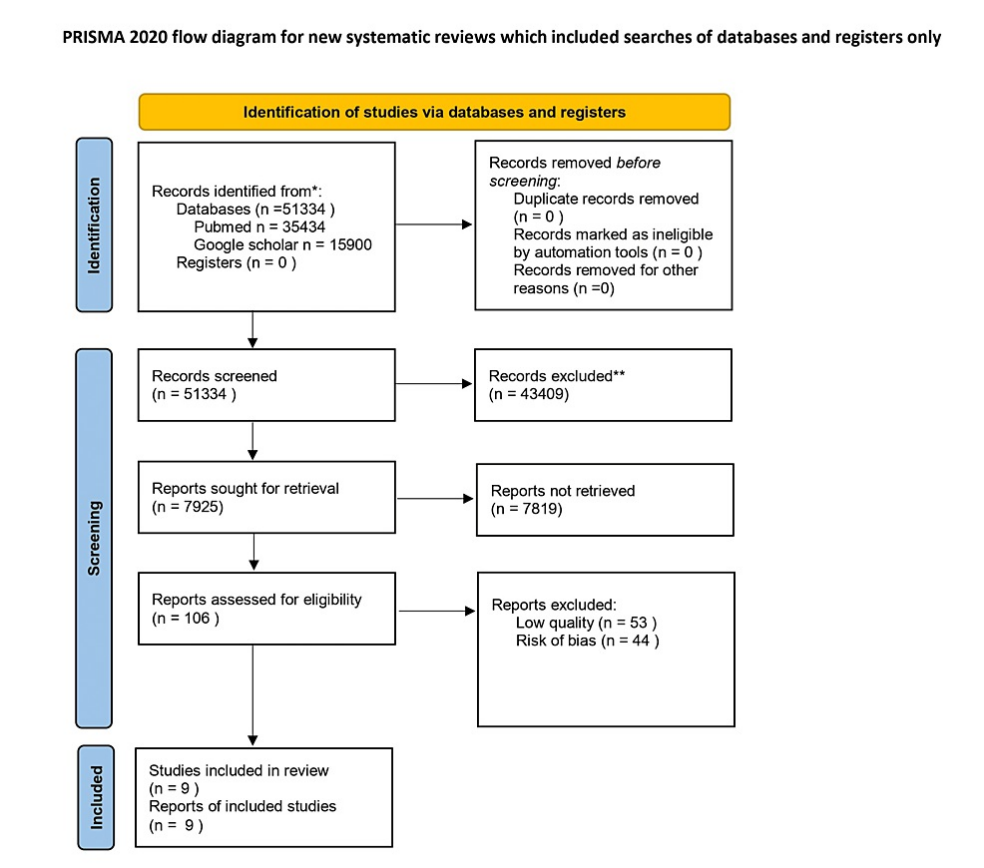
PRISMA flow diagram outlining the screening and selection process of articles obtained from different databases * the number of records identified from each database or register searched (rather than the total number across all databases/registers) ** the number of records were excluded by a human and by automation tools PRISMA - Preferred Reporting Items for Systematic Reviews and Meta-Analysis n - Number

The PRISMA flow chart depicting the search process and study selection is shown in Fig. 1. A total of 51355 studies were identified through our competitive search. There were no duplicates, and 43430 articles were omitted after reading the titles and abstracts because they did not fit the inclusion criteria. Finally, free full-text articles were retrieved of which only nine were included based on the inclusion, exclusion criteria, and also bias assessment.

Characteristics of the Included Studies

The characteristics of the included studies are shown in Table 2. The studies included in this review were published from 2010 to 2021. Our studies included systematic reviews, meta-analysis, and observational studies like cross-sectional and cohort studies. Five of the studies are observational studies, and three of them are systematic analysis and one is both systematic and meta-analysis.

Quality Assessment

Table 3 and Table 4 demonstrate the quality assessment of each paper. The Newcastle-Ottawa scale was used to assess quality for observational studies, and Amstar 2 was used to assess the quality for systematic and meta-analysis studies. Overall, all our included studies were of good quality, and less bias was noticed.

**Table 3:** Quality assessment by the Newcastle-Ottawa scale

References	Selection	Comparability	Exposure	Overall grade
Gibson-Smith et al. [13]	****	**	***	GOOD
Gregório et al. [14]	***	**	***	GOOD
Jacka et al. [15]	****	*	***	GOOD
Vafaei et al. [18]	****	*	***	GOOD
Weng et al. [17]	****	*	***	GOOD

**Table 4:** Quality assessment by the AMSTAR 2 checklist

Sl.No	AMSTAR 2 ITEMS	Dharmayani et al. [11]	Głąbska et al. [12]	Lassale et al. [16]	Quirk et al. [9]
		2021	2020	2018	2013
1.	Was an ‘a priori’ design provided?	No	Yes	No	No
2.	Was there duplicate study selection and data extraction?	Yes	Yes	Yes	Yes
3.	Was a comprehensive literature search performed?	Yes	Yes	Yes	Yes
4.	Was the status of publication (i.e. grey literature) used as an inclusion criterion?	Yes	Yes	Yes	Yes
5.	Was a list of studies (included and excluded) provided?	Yes	Yes	Yes	Yes
6.	Were the characteristics of the included studies provided?	Yes	Yes	Yes	Yes
7.	Was the scientific quality of the included studies assessed and documented?	Yes	Yes	Yes	Yes
8.	Was the scientific quality of the included studies used appropriately in formulating conclusions?	Yes	Yes	Yes	Yes
9.	Were the methods used to combine the findings of studies appropriate?	Yes	Yes	Yes	Yes
10.	Was the likelihood of publication bias assessed?	Yes	Yes	Yes	Yes
11.	Was the conflict of interest included?	Yes	No	Yes	Yes
	Total grade	HIGH	HIGH	HIGH	HIGH
	Total score out of 11	10	10	10	10
	Total percentage = 90.90%				

The Newcastle Ottawa quality assessment scale for observation studies is as follows:

Thresholds for converting the Newcastle - Ottawa scales to AHRQ standards ( good, fair, and poor):good quality: 3 or 4 stars in selection domain AND 1 or 2 stars in comparability domain AND 2 or 3 stars in outcome/exposure domain Fair quality: 2 stars in selection domain AND 1 or 2 stars in comparability domain AND 2 or 3 stars in outcome/exposure domain Poor quality: 0 or 1 star in selection domain OR 0 stars in comparability domain OR 0 or 1 stars in outcome/exposure domain. 

Discussion

This systematic review aimed to evaluate the association between dietary habits and depressive symptoms in young people, adults, and older people. We identified nine studies of which five are observational studies and four are systematic reviews. Studies included in this review were conducted in various countries. Our major aim was to find some correlation between dietary habits and depression.

Based on the present data, Dharmayani et al. highlighted the potential of fruit and vegetable consumptions and their association with depression in young people and adults but at the same time, they also emphasize that there is insufficient evidence regarding the effect of fruits and vegetables on lowering the risk of developing depression and depressive symptoms and they also stated the need for more evidence-based research to be conclusive of the results, while Glabska et al. argued that there is a link between adolescents diet of fruits and vegetable products and their mental health and also stated that green veggies, yellow vegetables, and fresh fruits were found to be particularly advantageous for the general mental health of teenagers, as did other fruits and vegetables [11,12].

The study by Dharmayani et al. is also a first of its kind in this topic, and different definitions of depression or depressive symptomatology exist because of inconsistency in outcome measures used, which may have had an impact on the findings as well. Both the studies were systematic reviews and showed that there is a definite potential for association between fruit and vegetable intake and depression, but still we need robust research on their outcomes to be conclusive [11,12].

When we take into the diet ingredients, Gibson-smith et al. argued that higher vegetable consumption was associated with reduced depression, anxiety, and fear intensity. Compared to healthy controls, higher consumption of non-refined grains was associated with reduced severity of depression and anxiety arousal, as well as reduced odds of having a current clinically diagnosed condition. Compared to moderate drinkers, non-drinkers had more severe depression and anxiety and were more likely to be currently depressed. Thus, these appear to be the key ingredients of the Mediterranean diet. The Mediterranean diet score revealed that a poor diet was associated with both a diagnosis of depression/anxiety and more severe symptoms. Total calorie consumption was linked to more severe anxiety, depression, and anxiety/depression symptoms, albeit the latter two were not statistically significant when multiple testing was used. Therefore, although there are strong correlations between diet and depression/anxiety, the impact of food groups on depression was modest for individual patients but may be clinically important for populations. Overall, the direction of food group association was as expected. Thus, a higher intake of non-refined grains, vegetables, fruits, potatoes, fish, and olive oil was associated with less severe depression or anxiety, whereas a higher intake of poultry and high-fat dairy products was associated with more severe depressive/anxiety disorder symptoms. Notably, larger intake of red and processed meat was associated with lower severity scores and the likelihood of a present disorder. Since the Mediterranean diet score had the highest relationships, it appears that the cumulative effect of nutrients from diverse food groups is linked to mental health [13].

The study conducted by Gregorio et al. among Portugal people discovered diet-vulnerable strata (males, young age, lower years of education, unemployment, part-time job, domestic working, and island dwellers). Furthermore, poor dietary practices were linked to depression symptoms when it included a lower frequency of soup, vegetables, fruit, fish, and milk/dairy, less water intake, and a higher frequency of meat consumption. Additionally, poor dietary practices are linked to other unhealthy lifestyle behaviors like inactivity, smoking, and alcoholic habits, reinforcing the premise that behavioral change programs should target many lifestyle domains. To improve the health of the vulnerable strata, health education and multiple health behavioral modification programs should be implemented quickly [14].

The data from the study by Jacka et al. showed a cross-sectional population-based investigation supporting the hypothesis of habitual diet quality and high-prevalence mental diseases. In contrast, a diet rich in processed and "unhealthy" foods (the western diet) was linked to an increased risk of psychiatric symptoms and disorders. A better food quality score was connected with less psychological symptoms. After adjusting for age, socioeconomic position, education, physical activity, and other lifestyle factors, the associations remained [15].

Jacka et al. highlighted the positive relationship between the western dietary pattern and depressive disorders after adjusting for total energy intake. This suggests that the absolute amount of harmful foods ingested is more important for mental health than the proportion of total diet. It was surprising that a modern food pattern was related to an increased risk of depression, rather than a decreased risk. A reverse causality effect may explain why younger, better-educated women with higher scores on the modern dietary component altered their diet to alleviate symptoms. Another argument is that conventional dietary components including vegetables, red meat, whole grains, and high-fat dairy items are especially relevant to the outcomes. This relationship may also be a type I mistake. We are investigating the effects of specific food components on mental health. The study by Lassale et al. found that there was a "significant association" between a lower risk of depression and following a Mediterranean diet more and a pro-inflammatory diet less [15,16]. 

Dietary patterns or feeding habits fluctuate from person to person, making it difficult to pinpoint the exact source of the diet that causes mood disorders. Hence, we need a specific plan to figure out the right dietary plan to tackle this problem. Quirke et al. argued that similar themes appeared while analyzing probable reasons for the conflicting findings across research. Despite the excellent methodological quality of most included studies, there was considerable variation in food quality evaluation, depression assessment, and study sample selection. Notable were the varying definitions of ‘healthy diet' and the measuring of diet quality and patterns [9].

In this field of study, the difficulty of measuring is acknowledged as a challenge because large measurement error reduces the ability to find correlations, which may be useful in explaining apparent inconsistencies. This prevented us from performing a meta-analysis. Similarly, the large range of depression measurements utilised may have hidden or diluted potentially substantial depression-diet quality correlations. There are significant clinical and public health repercussions if there is a genuine causal relationship between diet quality and depression that is concealed by methodological shortcomings. Eating well could not only help prevent depression, but it could also help individuals who already have it. Given that many patients do not react to pharmaceutical or psychological treatments, this is an area that need more research [9].

The study by Weng et al. was performed on boys and girls using main component analysis, the food frequency questionnaires revealed three eating patterns: snack, animal food, and traditional food. There was also a lot of confectionery and preserved fruit in the snack diet. Meat was consumed in many forms, such as fried or organ meat and processed meat. The customary diet included gruel, oats, whole grains, fresh yellow or red vegetables, fruit, and soy milk. The prevalence of depression, anxiety, and coexisting was 11.2%, 14.6%, and 12.6% respectively. Depression and anxiety were linked to girlhood, having an only child, low maternal and father educational levels, low family income, and inactivity. The highest tertiles scores in the snack dietary pattern were associated with higher odds of depression and anxiety disorders. The associations were strengthened by all adjustments for age, gender, and maternal and paternal education. In both unadjusted and adjusted models, traditional food was linked to lower chances of mental problems [17].

Vafaei et al. reported that malnutrition and depression are common among the elderly and can negatively impact their health and well-being. Detecting changes in primary care can improve the quality of life and reduce complications. Because malnutrition is multi-factorial and usually coexists with other issues, nutritional interventions should only be implemented as part of a comprehensive strategy. Inter-disciplinary efforts to increase nutrient intake and remove malnutrition risk factors should be encouraged. To prevent and/or treat elderly malnutrition, a multidisciplinary team of gerontologists, psychiatrist, anthropologists, clinicians, nutritionists, and other specialists is required [9,17,18].

## Conclusions

According to the findings of our study, there may be a significant connection between certain dietary behaviors and signs and symptoms of depression in people of all ages. Following a healthy diet, particularly one that incorporates vegetables and fruits, and avoiding a pro-inflammatory diet such as junk food, fast food, and a high intake of meat may lower the risk of developing depressive symptoms or clinical depression, according to the findings of our review. The present study reports that following a healthy diet, particularly one that incorporates vegetables and fruits, may lower the risk of developing clinical depression.

It is necessary to conduct additional research in order to address the particular features of nutrition that may be connected with the prevention of depression from manifesting itself. In addition, we recommend the execution of prospective cohort studies as well as randomized controlled trials in order to more thoroughly investigate the connection between diet and depression. The research that is currently being carried out has the potential to result in a number of outcomes, some of which include evidence-based policymaking and the addition of positive mental health outcomes to an already extensive list of reasons why people should prioritize maintaining a healthy diet.
